# GODEEEP-hydro: Historical and projected power system ready hydropower data for the United States

**DOI:** 10.1038/s41597-025-05097-3

**Published:** 2025-05-28

**Authors:** Cameron Bracken, Youngjun Son, Daniel Broman, Nathalie Voisin

**Affiliations:** 1 Pacific Northwest National Laboratory, Richland, Washington, USA; 2 Civil and Environmental Engineering Department, University of Washington Seattle, WA, USA

**Keywords:** Hydrology, Hydroelectricity

## Abstract

Hydropower is a critical electricity resource in the United States which, in addition to low-cost electricity generation, provides valuable ancillary grid services, and supports the integration of nondispatchable weather-dependent resources (e.g., wind and solar). Despite its value to the grid, there are very few comprehensive datasets available from which to study both historical and future impacts of climate, weather driven energy droughts, and integration of other weather driven generation. In this paper, we present a hydropower generation dataset covering 1,452 hydroelectric plants in the contiguous U.S. The dataset contains monthly and weekly hydropower generation estimates for both historical (1982–2019) and future (2020–2099) periods which includes 4 future climate scenarios. In addition, this dataset provides weekly and monthly constraints such as minimum and maximum power which are particularly useful in power system models which are used to study grid reliability, transmission planning and capacity expansion.

## Background & Summary

Hydropower is a critical electricity resource in the United States (U.S.) accounting for an average 6.63% of annual utility scale generation from 2013 to 2023.^1^ Hydropower can also provide a range of ancillary services such as load factoring, operating reserves, voltage support, and blackstart that are especially valuable as low-cost non-dispatchable resources are added to the grid.^2^ Despite the importance of hydropower to the grid, there are limited comprehensive datasets available from which to study both historical and future impacts of climate, weather driven energy droughts, and integration of other weather driven generation. In this paper, we present a hydropower generation dataset covering 1,452 hydroelectric plants in the contiguous U.S comprised of both federal and privately owned facilities. The dataset contains monthly and weekly hydropower generation estimates for both historical (1982–2019) and future (2020–2099) periods, the latter containing 4 different climate scenarios.

Power system models such as production cost models (PCMs) represent the power system as an optimization problem where energy demands are met with both dispatchable resources such as natural gas and non-dispatchable resources such as wind and solar. These models often treat hydropower as a dispatchable resource which requires operational constraints such as power targets, minimum generation, maximum generation, and ramping rates which serve as approximations of true hydropower operations. The data presented here includes these operational constraints and so can be readily used to represent nearly all existing conventional U.S. hydropower generation in power system models.

**Fig. 1:**
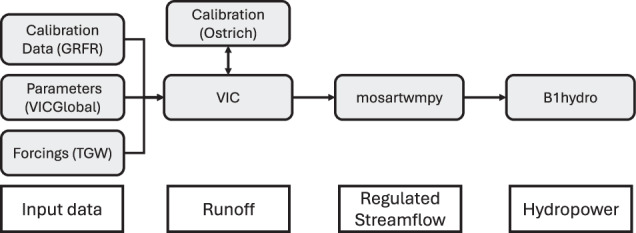
Modeling chain used to develop hydropower estimates. The grey boxes indicate models or datasets, and the white boxes indicate the major output from each step of process.

The development of hydropower generation and power constraints requires a series of models and data including meteorology, hydrology, routing, water management, and hydropower (Fig. 1). A distributed hydrology model takes meteorology data as input and computes gridded runoff, based on the calibrated hydrologic parameters. The runoff data is passed to a routing model which develops natural streamflow estimates within a river channel network developed on a uniform 1/8th degree grid. At certain grid cells where dams are present, the routing model must take into account human management, including reservoir operations and water demands. The final model in the chain converts regulated streamflow to hydropower. The following sections describe the models in greater detail.

## Methods

### Meteorology Data

In this study we used perturbed thermodynamics experiments^3,4^ (https://tgw-data.msdlive.org/) as meteorological forcing. The forcing data is a 1/8th degree dynamically downscaled product which contains both historical data and future projections over the contiguous United States (i.e. lower 48 states), southern Canada, and northern Mexico. The dynamically downscaled data is produced by initializing a WRF^5^ model using ERA5^6^ boundary conditions. The future projections are developed by replicating the historical period (1980–2019) twice in the future (2020–2059, 2060–2099) while applying a warming signal that is derived from groups of Coupled Model Intercomparison Project (CMIP) 6 models.^4^ The warming scenarios are labeled as rcp45cooler, rcp45hotter, rcp85cooler, and rcp85hotter which represent a range of warming signals derived from climate models using the representative concentration pathway (RCP) 4.5 and 8.5 emissions scenarios.

### Hydrology Model

For hydrologic modeling we use the variable infiltration capacity (VIC) model^7,8^ (https://vic.readthedocs.io/en/master/). VIC is a commonly used model for large scale distributed hydrologic modeling studies. Parameters are obtained from the VICGlobal^9^ dataset which contains vegetation and soil parameters on a 1/16th degree grid. We calibrate the parameters against the Global Reach-level River Flood Reanalysis data^10^ which is a global dataset of 1/20th degree runoff. Calibration is conducted for 1981–2000 at 1/16th degree resolution on a grid cell by grid cell basis. For automatic calibration we use the dynamically dimensioned search (DDS) algorithm^11^ through the Optimization Software Toolkit for Research Involving Computational Heuristics (OSTRICH) framework^12^ (https://doi-bor.github.io/ostrich/). The DDS algorithm is designed to provide a reasonably optimal solution within a limited computational budget, here we used 100 iterations of the DDS algorithm as testing indicated that more iterations provided marginal improvement to the objective function value. For the objective function we used the Kling-Gupta Efficiency (KGE) metric of monthly observed runoff as it provides a good balance between low and high runoff conditions. The KGE metric is described further in the validation section. Table 1 shows the calibration parameters and the ranges which are selected based on previous hydrologic studies using the VIC model^9,10,13,14^.

**Table 1:** VIC parameters optimized in the auto-calibration process with the min and max allowed parameter values.

Parameter	Description	Unit	Min	Max
b	Shape Parameter for Variable Infiltration Capacity Curve	—	0.01	0.8
*D*_*m*_	Maximum Baseflow Velocity	mm/day	1	30
*D*_*s*_	Fraction of Dm for Linear Baseflow Curve	Fraction	0	1
*W*_*s*_	Fraction of Maximum Soil Moisture for Linear Baseflow Curve	Fraction	0.5	1
*d*_2_	Thickness of Intermediate Soil Layer	m	0.1	2
*d*_3_	Thickness of Bottom Soil Layer	m	0.1	2
Expt_2_	Brooks-Corey Exponent for Intermediate Soil Layer	—	8	30
Expt_3_	Brooks-Corey Exponent for Bottom Soil Layer	—	8	30

### Routing and Water Management Model

Routing is conducted at a 1/8th degree scale by the mosartwmpy model^15^ (https://mosartwmpy.readthedocs.io/en/latest/), a Python implementation of the MOSART-WM model,^16,17^ which is part of the Energy Exascale Earth System Model (E3SM) (https://e3sm.org/). Routing alone produces gridded natural streamflow estimates but water management is required to develop estimates of regulated streamflow, storage, inflow, and outflow for hydropower projects, which mosartwmpy produces through the use of data driven reservoir operation rules^18^. Hydropower projects were mapped to the 1/8th degree grid as part of the 9505 federal assessment of hydropower^19^.

### Hydropower Model

The final model in the chain takes the regulated streamflow values produced by mosartwmpy and generates weekly and monthly hydropower estimates, which we call B1hydro. At every hydropower plant, B1hydro models the power generation as a linear regression model with the form:

1 $$\begin{array}{l}{P}_{t}={\beta }_{P,1}{P}_{t-1}+\ldots +{\beta }_{P,n}{P}_{t-n}+\\ \hspace{4.99878pt}\quad {\beta }_{O,0}{O}_{t}+{\beta }_{P,1}{O}_{t-1}+\ldots +{\beta }_{O,n}{O}_{t-n}+\\ \hspace{4.99878pt}\quad {\beta }_{I,0}{I}_{t}+{\beta }_{I,1}{I}_{t-1}+\ldots +{\beta }_{I,n}{I}_{t-n}+\\ \hspace{4.99878pt}\quad {\beta }_{S,0}{S}_{t}+{\beta }_{S,1}{S}_{t-1}+\ldots +{\beta }_{S,n}{S}_{t-n}+{\varepsilon }_{t}\end{array}$$

where *P*_*t*_ is the power at time *t*, *O* denotes the outflow, *I* denotes the inflow, *S* denotes the storage, which are outputs from the mosartwmpy model, *β*_*i*,*j*_ are the regression parameters, and *ε*_*t*_ is the normally distributed error term. The lag parameter *n* is set to 12 and 52 for the monthly and weekly model respectively to account for annual hydrologic variability.

The data used to calibrate the regression parameters is the HydroWIRES B1 data^20,21^ (https://github.com/HydroWIRES-PNNL/B1-data), which contains weekly and monthly hydropower estimates that are disaggregated from U.S. Energy Information Administration (EIA) annual data^22^. The data is available for 2001–2019 which is used as the calibration period to develop both the historical and future hydropower data at every available hydropower plant location. Of the 1492 plants in the HydroWIRES B1 data, 1452 are included in the GODEEEP-hydro dataset, with the 40 plants excluded due to records that were too short (less than 2 years) or containing all zero values (i.e. decommissioned). The entire historical period (1982–2019) is included in the final dataset to provide (1) validation with observations and other derived datasets, (2) an extension the historical record beyond what is available in the HydroWIRES B1 data, and (3) a consistent record of hydropower that is coincident with other datasets derived from the perturbed thermodynamics experiment data (e.g.^23^).

In addition to total generation over the weekly or monthly period, the B1hydro model provides minimum and maximum power generation of the period and the average daily operational range (ador), which are directly useful in power system models. These values are defined as:

2 $$\begin{array}{rcl}{P}_{max,t} & = & {P}_{t}+{a}_{max}({P}_{np}-{P}_{t})\\ {P}_{min,t} & = & {a}_{min}{P}_{t}\\ {P}_{ador,t} & = & {a}_{ador}\left({P}_{max,t}-{P}_{min,t}\right)\end{array}$$

where *P*_*max*,*t*_ and *P*_*min*,*t*_ are the max and min allowed power generation at time *t*, *P*_*ador*,*t*_ is average daily operational range at time *t*, *P*_*t*_ is the average power generation at time *t*, *P*_*np*_ is the nameplate capacity of the plant, and *a*_*max*_, *a*_*min*_, and *a*_*ador*_ are parameters with values between 0 and 1 which can be derived from hourly power generation data.

Hourly hydropower generation is usually proprietary and business sensitive and therefore not publicly available. The Army Corps of Engineers Northwestern Division, which includes the Columbia River Basin, is one exception where historical hourly generation data from most federally-operated hydropower facilities is published on the Dataquery platform (https://www.nwd-wc.usace.army.mil/dd/common/dataquery/www/). The B1hydro model uses this hourly generation data to estimate the min and max power and ador at every hydropower plant in this study by assuming that *a*_*max*_, *a*_*min*_, and *a*_*ador*_ are equal to the average parameter values from all the hydropower facilities on the Dataquery platform. This approximation allows for reasonable constraints to be developed for power system models given the scarcity of publicly available hourly hydropower data.

### Future Simulations

The four future climate scenarios in the TGW data (rcp45cooler, rcp45hotter, rcp85cooler, and rcp85hotter) are used to develop future hydropower simulations from 2020–2099. The calibrated VIC model is used to produce future runoff simulations for each scenario, which are then run through mosartwmpy, and finally the calibrated B1hydro model is used to produce monthly and weekly hydropower generation estimates.

## Data Records

The data is available from Zenodo^24^ (10.5281/zenodo.13776944).

The data is split into 10 data files, weekly and monthly data for each future scenario and the historical period. Each file has the naming convention <scenario>_ <monthly/weekly>.csv where <scenario> can be either “historical”, “rcp45cooler”, “rcp45hotter”, “rcp85cooler”, or “rcp85hotter” and <monthly/weekly> refers to the timestep of the data. Each of the data files has the following columns:

**datetime** The datetime stamp of the current timestep

**eia**_**id** An integer value with the EIA plant code that represents the facility

**plant** The name of the facility according to the EIA

**power**_**predicted**_**mwh** The total energy generated over the period in MWh, aka the energy target

**n**_**hours** The number of hours in the period, useful for converting between power and energy

**p**_**avg** Average power generation for the period

**p**_**max** Maximum allowable power generation for the period

**p**_**min** Minimum allowable power generation for the period

**ador** Average daily operational range for any given day in the period

**scenario** The name of the scenario, either “historical”, “rcp45cooler’, “rcp45hotter”, “rcp85cooler”, or “rcp85hotter”

Also included is the metadata file godeeep_hydro_plants.csv which contains metadata for each hydropower plant that is included in the dataset. Each row in this file refers to one hydropower facility. This file has the following columns:

**eia**_**id** An integer value with the EIA plant code that represents the facility

**plant** The name of the facility according to the EIA

**mode** Either “Storage” or “RoR” indicating if the plant is primarily operated as a storage or run-of-river facility

**state** Two letter U.S. state name

**lat** Latitude of the facility

**lon** Longitude of the facility

**nameplate**_**capacity** The total nameplate capacity of the facility according to the EIA

**nerc**_**region** Four letter code for the NERC region of the facility

**ba** Balacing authority of the facility

**max**_**param** Value of the *a*_*max*_ parameter from Equation (2) used to derive p_max

**min**_**param** Value of the *a*_*min*_ parameter from Equation (2) used to derive p_min

**ador**_**param** Value of the *a*_*ador*_ parameter from Equation (2) used to derive ador

**huc2** Two digit hydrologic unit code (HUC) which contains the facility

## Technical Validation

### Hydrology model validation

**Fig. 2:**
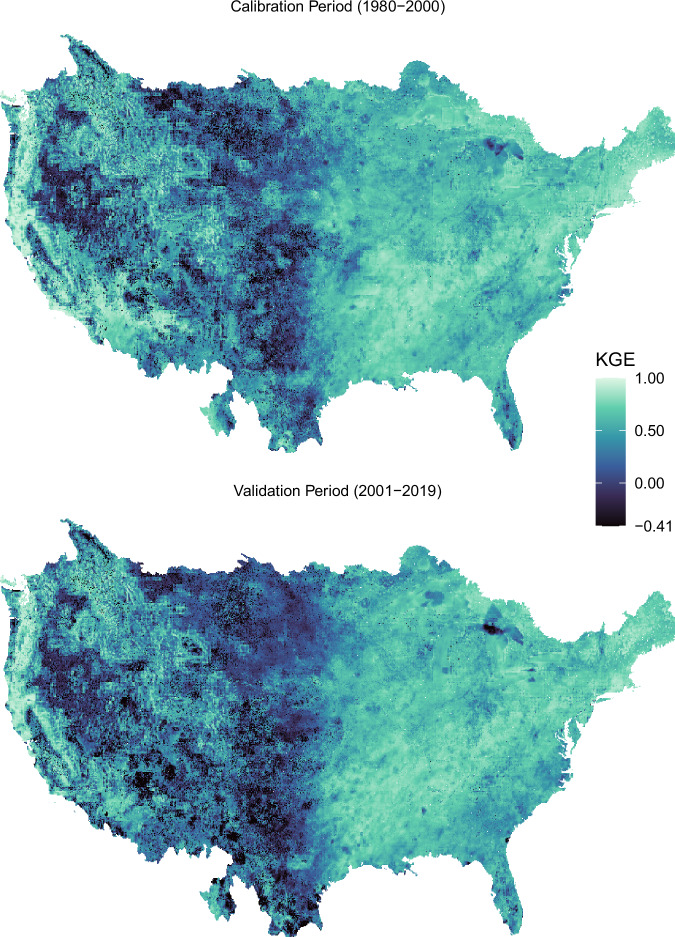
KGE values for the calibrated VIC model in the calibration period (top) and the validation period (bottom).

The VIC hydrology model is calibrated at a monthly timestep for the period 1981–2000, and validated for 2001–2019, with the period 1979–1980 used as spin-up. We use the KGE metric to assess model performance^25^ on simulating runoff. KGE is a commonly used metric in hydrology where any value greater than −0.41 indicates performance better than the mean^26^. Figure 2 shows the calibration results for the study region. The calibration results are consistently above −0.41 and frequently much higher. The calibration results align well with previous CONUS-wide calibration efforts,^27^ with the best performance on the east and west coast and lower performance in the Midwest region east of the Rocky Mountains. The validation period has lower performance than the calibration period, which is to be expected, but overall the periods are similar which is encouraging.

### Hydropower model validation

**Fig. 3:**
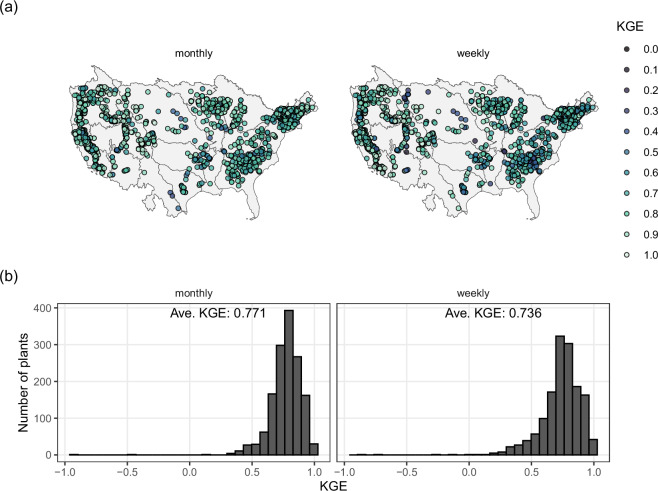
KGE values from the B1hydro model for all modeled hydropower plants for both monthly and weekly data.

The first validation of the B1hydro model is designed to test the regression model using drop-one-year cross validation. In this procedure one year of data is dropped and the other years are used to predict the missing generation data. Using this approach on every year of data provides a complete record from which to assess the out-of-sample performance. Figure 3 shows a histogram of the KGE of all plant level hydropower models that are part of the B1hydro model. The performance is generally good, with only 2 out of 1,452 plants having performance less than −0.41.

**Fig. 4:**
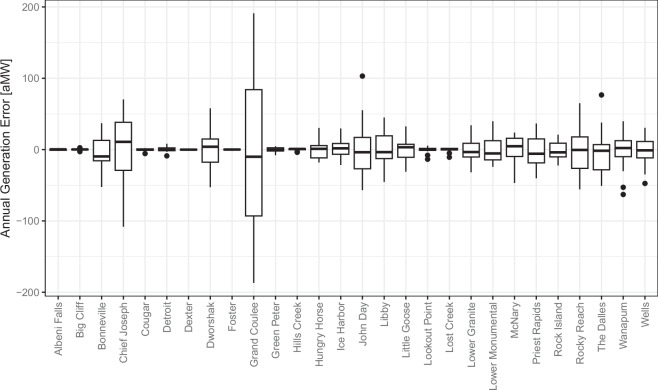
Annual verification of B1hydro predictions against generation reported by the Army Corps of Engineers Northwestern Division for Columbia River Basin hydropower plants.

Additionally, we validate the B1hydro model against observed hydropower data in the Columbia River Basin. Figure 4 shows boxplots of the difference in the annual generation between observed annual hydropower and the B1hydro model output. The greatest error of about 200 aMW is seen at Grand Coulee which has the highest hydropower generation of any plant in the region. In general, the error is proportional to the nameplate capacity of the hydropower plant and tends to bracket 0, indicating reasonable annual performance with no annual bias in the B1hydro model.

### Validation against 9505 data

The 9505 assessment is the Department of Energy funded assessment of the relationship between climate and hydropower in the U.S. (https://www.energy.gov/eere/water/hydropower-climate-change-assessment). One outcome of the 9505 assessment is the development of a hydropower dataset that uses a selection of hydrologic models, hydropower models, forcing datasets.^28^ Here we compare with two hydrology models, Precipitation-Runoff Modeling System (PRMS)^29^ and VIC, two hydropower models, wmpy-power (WMP) and WRES^28^, and one forcing dataset, Livneh^30^. To facilitate an accurate comparison, we have only compared the hydropower plants that are simulated by both datasets.

**Fig. 5:**
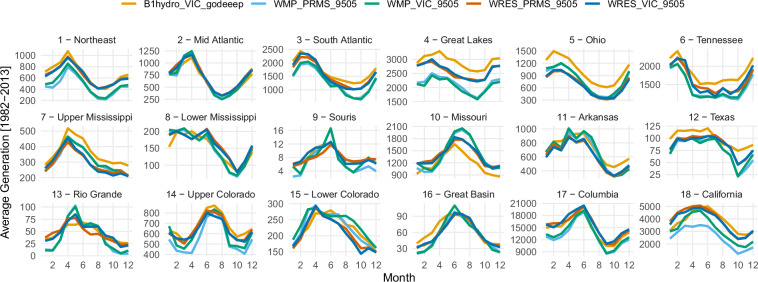
Monthly comparison of average total generation for each HUC2 basin in the contiguous U.S. Included in this comparison is the godeeeep_hydro data (B1hydro_VIC_godeeep), and four hydropower datasets that are part of the 9505 assessment, the wmpy-power model using the PRMS hydrology model (WMP_PRMS_9505), the wmpy-power model using the VIC hydrology model (WMP_VIC_9505), the WRES model using the PRMS hydrology model (WRES_PRMS_9505), and the WRES model using the VIC hydrology model (WRES_VIC_9505).

Figure 5 shows the average total monthly generation for each HUC2 in the contiguous U.S. The hydropower estimates from GODEEEP-hydro are generally in line with the 9505 estimates lending confidence to the methodology presented here. Some notable differences occur in the Great Lakes and Ohio basins where GODEEEP-hydro is higher than the 9505 models. This may be due to differences in the representation of hydropower between the U.S. and Canada.

## Usage Notes

The data is provided in csv files which should be readable in any modern software package. Each row of data in every file data file represents one timestep (either 1 month or 1 week). Some metadata is provided in each data row such as the EIA id, plant name and scenario name. If desired, the full set of metadata from godeeep_hydro_plants.csv can be joined to any data file using the eia_id column.

A companion dataset and paper^31^ providing hydropower data and PCM constraints for western Canada is available.

## Data Availability

All code to develop the dataset is available in Bracken and Son, 2025^32,33^.
